# Brugada ECG pattern precipitated by acute pneumonia: a case report

**DOI:** 10.1186/1757-1626-2-73

**Published:** 2009-01-21

**Authors:** Salaheldin Abusin

**Affiliations:** 1 Department of Medicine, John H. Stroger Hospital of Cook County, Chicago, IL, USA

## Abstract

Brugada type 1 ECG pattern is the hallmark for the diagnosis of Brugada syndrome which is a cause of sudden death due to ventricular arrhythmias. We present a case of a previously healthy young man who was admitted with productive cough with greenish phlegm and right-sided chest pain which was subsequently diagnosed as acute pneumonia. A routine ECG was done as part of his evaluation and showed Brugada ECG type 1 pattern. He was treated with antibiotics and on follow up his ECG was normal. In this report we present this increasingly described phenomenon and briefly review the literature.

## Background

Brugada type 1 ECG pattern is the hallmark for the diagnosis of Brugada Syndrome which causes sudden cardiac arrest. It is a distinctive pattern of pseudo RBBB, and persistent ST elevation in V1 to V3. In the acute setting, it is important to differentiate it from the other more common causes of ST elevation namely ST elevation Myocardial Infarction and pericarditis.

## Case presentation

**Figure 1:**
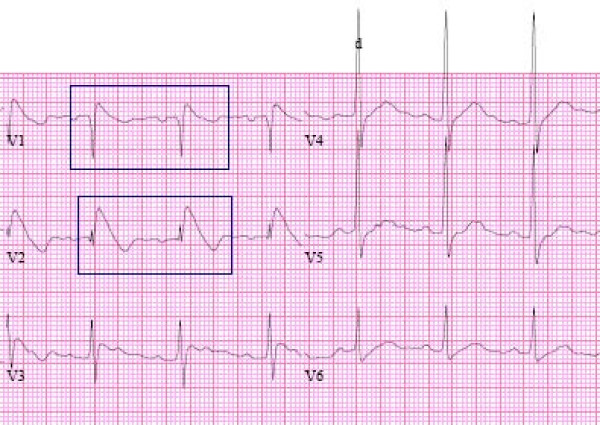
**Brugada ECG pattern**.

A 49-year-old Male presented to our institution with 4-days history of productive cough with greenish phlegm, right-sided chest pain worse on inspiration, fever and sweats. On physical examination, he was febrile with a temperature of 102°F, heart rate of 92, Blood pressure of 143/88. He had rales in the lower lobe of the right lung; the rest of his physical examination was normal and did not reveal a friction rub. Chest radiograph showed right lower lobe consolidation, he was diagnosed with community-acquired pneumonia and started on antibiotic therapy. An ECG was done (Figure 1); it revealed elevated ST segment "coved type" classical for Type 1 Brugada ECG. It did not show any changes suggestive of pericarditis namely, depression of the PR interval or diffuse saddle shaped ST elevation.

On further questioning the patient did not have any history of cardiac arrhythmias, syncope, past history of unexplained syncope, or family history of sudden death. Echocardiogram revealed a structurally normal heart with a normal ejection fraction, with no evidence of pericardial effusion. He had no rise in cardiac enzymes. He was discharged on oral antibiotic therapy. At one week follow up, the patient had recovered from pneumonia, and his ECG returned to normal.

Given his history the patient was considered low risk for an arrhythmic event and electrophysiological studies (EPS) were not pursued, he continues to follow up in the outpatient clinic.

## Discussion

Brugada ECG pattern can occur spontaneously or precipitated by a sodium channel blocker, e.g. flecainide, other medications include beta blockers, tricyclic antidepressants and lithium. Other precipitants include fever, hyperkalemia, hypokalemia, hypercalcemia, cocaine and alcohol. This case represents the second report in the literature of acute pneumonia precipitating Brugada ECG pattern [1].

The diagnosis of Brugada syndrome requires, in addition to the Brugada ECG pattern, one or more of 7 criteria [2]: ventricular fibrillation, self terminating polymorphic ventricular tachycardia, family history of sudden cardiac death at age less than 45 years, Brugada ECG pattern in a family member, electrophysiological inducibility of VT, unexplained syncope suggestive of a tachyarrhythmia, and nocturnal agonal respiration.

The value of EPS in risk stratification in patients with Brugada ECG pattern has been thrown into doubt in a recent meta-analysis of 15 studies [3]. It did not show that inducibility of sustained VT during EPS predicted the occurrence of further VTs during follow-up.

Moreover, a recent cohort study of incidentally found Brugada ECG pattern during a routine health examination, has shown no increased cardiovascular or all cause mortality in patients with Brugada ECG pattern. compared to those without the pattern, even after adjustment for cardiovascular risk factors. [4]

The specific treatment for patients with Brugada syndrome who are symptomatic or have family history of sudden death is prevention of sudden cardiac death by implantation of a cardioverter-defibrillator (ICD) [5].

## Conclusion

This case illustrates an increasingly seen phenomenon of Brugada ECG pattern precipitated by fever. It is important to recognize as select patients will require ICD placement to prevent sudden cardiac death.

## Abbreviations

ECG: electrocardiogram; VT: ventricular tachycardia

## Consent

Written informed consent was obtained from the patient for publication of this case report and accompanying images. A copy of the written consent is available for review by the Editor-in-Chief of this journal.

## Competing interests

The author declares that they have no competing interests.

## Authors' contributions

SA analyzed and interpreted the patient data, wrote the manuscript. He read and approved the final manuscript.
